# Modeling the evolution of a classic genetic switch

**DOI:** 10.1186/1752-0509-5-24

**Published:** 2011-02-05

**Authors:** Christos Josephides, Alan M Moses

**Affiliations:** 1 Departments of Cell & Systems Biology and Ecology & Evolutionary Biology, University of Toronto, 25 Willcocks Street, Toronto, ON, M5 S 3B2, Canada

## Abstract

**Background:**

The regulatory network underlying the yeast galactose-use pathway has emerged as a model system for the study of regulatory network evolution. Evidence has recently been provided for adaptive evolution in this network following a whole genome duplication event. An ancestral gene encoding a bi-functional galactokinase and co-inducer protein molecule has become subfunctionalized as paralogous genes (*GAL1 *and *GAL3*) in *Saccharomyces cerevisiae*, with most fitness gains being attributable to changes in *cis-*regulatory elements. However, the quantitative functional implications of the evolutionary changes in this regulatory network remain unexplored.

**Results:**

We develop a modeling framework to examine the evolution of the GAL regulatory network. This enables us to translate molecular changes in the regulatory network to changes in quantitative network function. We computationally reconstruct an inferred ancestral version of the network and trace the evolutionary paths in the lineage leading to *S. cerevisiae*. We explore the evolutionary landscape of possible regulatory networks and find that the operation of intermediate networks leading to *S. cerevisiae *differs substantially depending on the order in which evolutionary changes accumulate; in particular, we systematically explore evolutionary paths and find that some network features cannot be optimized simultaneously.

**Conclusions:**

We find that a computational modeling approach can be used to analyze the evolution of a well-studied regulatory network. Our results are consistent with several experimental studies of the evolutionary of the GAL regulatory network, including increased fitness in *Saccharomyces *due to duplication and adaptive regulatory divergence. The conceptual and computational tools that we have developed may be applicable in further studies of regulatory network evolution.

## Background

Regulatory networks are known to underlie many biological processes, and therefore their characterization and analysis forms a central focus of systems biology [1-4]. Despite their importance, relatively little is known about how regulatory networks are formed during evolution and shaped by natural selection.

One of the best studied regulatory networks in molecular biology is the "GAL network", which is responsible for the inducible metabolism of galactose in budding yeast. In addition to being extremely well-characterized in *S. cerevisiae *[5-7] it has also been the subject of a number of quantitative modeling efforts [8-11] and evolutionary studies, which have revealed many interesting patterns of regulatory network evolution [12-15]. Perhaps most general of these evolutionary paradigms is the duplication and divergence of function of an ancestral bi-functional gene. Before the whole genome duplication (WGD), which occurred along the lineage leading to *S. cerevisiae *[16,17], the GAL network is thought to have employed the Gal1/3 protein to perform both an enzymatic and regulatory function. This bi-functional protein has been retained in species that diverged before the WGD [18]. Following the WGD, the two gene copies of *GAL1/3 *were converted to a highly inducible enzyme (*GAL1*) [19] and a weakly inducible regulatory protein (*GAL3*) [20] in post-WGD species. Recent work has demonstrated that these changes confer a growth advantage in galactose [21], and suggested that gene duplication and subsequent neofunctionalization represented an example of how a regulatory network can overcome an adaptive conflict, whereby the network cannot improve in one aspect without impairing function in another.

**Figure 1:**
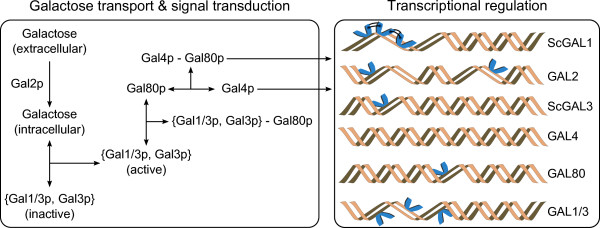
**Schematic of the GAL regulatory network model**. A transcriptional activator protein, Gal4p, binds a consensus upstream activating sequence (UAS) in the promoter region of structural and regulatory genes promoting their expression. In the absence of galactose, however, Gal4p forms a complex with the Gal80p repressor molecule, which masks its C-terminal activation domain, preventing the recruitment of transcriptional machinery. Galactose is imported from the environment by non-specific hexose transporters, as well as by highly-specific Gal2p permeases, and activates the co-inducer Gal3p molecule or the bi-functional Gal1/3p if it is present in the network. The activated form of Gal3p (and/or Gal1/3p) can then bind and sequester Gal80p, relieving the inhibition of gene expression and permitting induction of the GAL cluster. *GAL1 *has a core of three UASs arranged in an in-phase helical manner. This configuration allows cooperative interactions (black connecting lines in figure) between adjacently bound activating (Gal4p) and inhibiting (Gal4p-Gal80p complex, not shown) protein pairs, which enhance gene expression or inhibition in induced or uninduced conditions respectively. The promoter region of *GAL1/3 *also contains this core, but, unlike *GAL1*, these sites are arranged in an anti-phase helical configuration which does not permit cooperative interactions. *GAL3 *has retained only one UAS, resulting in decreased inhibition in uninduced conditions and a relatively small induction in the presence of galactose. In addition to the changes in the number and arrangement of *cis*-regulatory elements, differences also exist between the intrinsic strengths of ancestral and extant promoters (see **Table 4**). Note that cooperative interactions between the two UASs on *GAL2 *are permitted [5], and that the 4^th ^UAS on *GAL1 *(and *GAL1/3*) is not included in the model after indications that it may not be as important for gene expression as the other UASs [42].

Here we set out to explore the consequences of evolutionary changes in the GAL network using a model of the regulatory network (Figure 1) to relate specific sequence changes to changes in quantitative function. Starting with experimentally characterized regulatory differences between the pre- and post-WGD GAL genes (see Methods), we inferred an ancestral organization of the regulatory network (see Results). Simulations of the ancestral and *S. cerevisiae *networks show that gene duplication and specialization lead to elevated gene expression in the presence of galactose, and decreased gene expression in the absence of galactose, thus leading to an improved switch-like system in *S. cerevisiae*. We then use the model to explore the significance of the order in which a set of maximally parsimonious evolutionary events separating a post-WGD ancestor and *S. cerevisiae *occur, and find important consequences for the function and evolution of the switch. We introduce the idea of evolutionary paths in the space of possible regulatory networks and develop quantitative measures to compare paths. We find that there are evolutionary paths in the lineage to *S. cerevisiae *that optimize particular features of their constituent network intermediates, some of which have been shown to be directly related to fitness. Perhaps more importantly, we find that it does not seem possible to optimize all network features in any single path.

## Results

### A hybrid stochastic-deterministic modeling framework for examining the evolution of the GAL regulatory network

In order to explore the functional consequences of evolutionary changes in the GAL network, we sought a quantitative approach to relate these molecular changes to changes in network operation. Because the evolutionary events of interest include variations in gene copy-number, changes in protein function, as well as changes in the number and spacing of *cis*-regulatory sequences, we required a modeling framework in which we could incorporate all these features and relate them to network behavior.

To model transcription/translation we modified and implemented a general physicochemical model of transcription regulation [22,23] in a stochastic context. This allowed us to vary the regulatory features of promoters driving the GAL genes and hence to predict the effects of evolutionary changes in the *cis-*regulatory sequence of a particular promoter. To model changes in gene copy number, we simply changed the corresponding probability of transcription, for example, to model a gene duplication event, we multiplied the probability of that gene's transcription by two. Because of the difference in time scales and species numbers between transcription/translation reactions and protein-protein/galactose interactions [24], we modeled the latter using a system of ordinary differential equations. In order to model the effect of evolutionary changes in protein function, we assigned different sets of reaction possibilities to new molecular species created during the evolution of the network.

**Figure 2:**
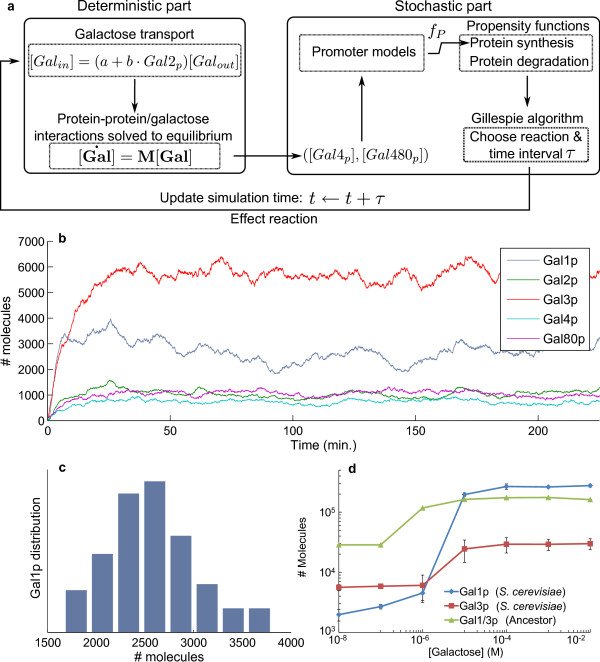
**Simulation algorithm and results**. **a**. The deterministic part models galactose transport and protein-protein/galactose interactions. Steady-state concentrations are used to update the system's components but, regardless of the time taken to reach equilibrium, simulation time is not advanced. The state of the system ([Gal4p], and [Gal4-80p], in particular) is then used to update the stochastic propensity functions for protein synthesis via the statistical-mechanical promoter models. The Gillespie algorithm selects a reaction channel and a time, *τ *containing no protein synthesis or degradation reactions, which is used to update the simulation time. The system's components are updated according to the selected reaction, and the algorithm re-iterates. **b**. Typical time-series data from a single simulation of the GAL network in *S. cerevisiae *in very low galactose (10 ^-7 ^M). **c**. Gal1p equilibrium protein distributions obtained from 100 simulations of the *S. cerevisiae *GAL network in very low galactose. **d**. Induction-response curves were constructed from equilibrium protein distributions in increasing concentrations of galactose. Bars indicate +/- standard deviation. Prior to the WGD, the bi-functional Gal1/3p molecule served as the network's co-inducer and galactokinase.

Hybrid approaches [25-28] such as this permit the inclusion of gene expression noise [29,30], multiple time scales, and variable system mass in biological models while making simulations computationally feasible. Briefly, each step of the hybrid simulation algorithm consists of a stochastic part for the slow transcription/translation/degradation processes, and a deterministic part for the fast molecular interactions which is solved to equilibrium (Figure 2a, see Methods section for a detailed description of the simulation algorithm).

Model parameters were chosen to reproduce the quantitative operation of the GAL network in *S*. *cerevisiae *([5,6,9,21], see Methods for details of parameter choice and parameter sensitivity). Typical traces from simulations are shown in Figure 2b. Protein distributions were obtained at equilibrium from 100 independent simulations of each variant network at constant extracellular galactose concentrations in the range [10^-8^, 10^-1^] M in the absence of glucose (Figure 2c). The induction-response curve for *S. cerevisiae *in increasing concentrations of galactose displays the characteristic switch-like response (Figure 2d) and fold-inductions in gene expression are consistent with previously published experimental data (Table 1 in Methods).

**Table 1:** Simulation and literature results for the GAL network in *S. cerevisiae*

		Simulation		Literature
**Gene**	**# molecules (uninduced)**	**#molecules (induced)**	**Fold-increase**	**Fold-increase**

***GAL1***	2567 +/- 494	270810 +/- 20757	105	100-1000 [5,6,9]

***GAL2***	1010 +/- 329	119130 +/- 8467	118	100-1000 [5,6]

***GAL3***	5791 +/- 254	29410 +/- 1810	5	3-5 [5,6]

***GAL4***	755 +/- 75	750 +/- 66	1	1

***GAL80***	1101 +/- 94	6390 +/- 449	6	5-10 [5,6]

Mean numbers of molecules +/- standard deviation is reported from equilibrium protein distributions of 100 simulations in very low galactose (uninduced) or very high galactose (induced).

### Reconstructing the operation of an inferred ancestral network

*GAL1 *and *GAL3 *are paralogous genes which have diverged after a WGD event in the lineage leading to *S. cerevisiae *from a common bi-functional ancestor, *GAL1/3*, which serves as both the galactokinase and co-inducer molecule. It has recently been shown that the fitness of cells growing in galactose is related to the phenotype and operation of the GAL network. The *cis*-regulatory evolution of *GAL1 *and *GAL3 *has led to a more efficient genetic switch by subfunctionalizing the processes and regulation of galactose phosphorylation and induction [21].

To study the effects of these changes on the network's quantitative function we inferred an ancestral form of the network by removing the *GAL1 *and *GAL3 *genes from the *S. cerevisiae *network and substituting a bi-functional *GAL1/3 *gene driven by the ancestral promoter for *KlacGAL1 *[21]. We have assumed that the other ancestral regulatory genes have remained unchanged in function and copy-number compared to *S. cerevisiae*.

We find that, relative to the inferred ancestor, the number of Gal1p (galactokinase) molecules in *S. cerevisiae *is increased in high galactose and reduced in very low galactose, which implies a more switch-like response in (105 fold in *S. cerevisiae *vs. 6 fold in the ancestor). At the same time, the number of Gal3p (inducer) molecules is decreased in all conditions relative to the ancestral protein (e.g., at very low galactose, there are ~5000 molecules in *S. cerevisiae *vs. ~27000 in the ancestor, Figure 2d). Thus, a single bi-functional gene seems to perform poorly in controlling the enzymatic and regulatory aspects of the network, consistent with experimental results [21].

### The effect of an evolutionary change on the quantitative function of the GAL network depends on the network's evolutionary history

Having established the quantitative differences in network function between the ancestral and extant GAL network in *S. cerevisiae*, we next sought to explore the significance of the order of evolutionary events after the WGD on network operation.

Five events were considered: the regulatory and functional divergence of the two *GAL1/3 *copies, and the loss of the *GAL2*, *GAL4*, and *GAL80 *gene duplicates, according to the principle of maximum parsimony [31]. Since we know that there was an ancestral genome duplication event, we expect each gene to have been at two copies in some ancestor. We then make the maximally parsimonious assumption that each other gene was lost once. This is the minimum number of evolutionary changes that could have occurred, though not necessarily the scenario that was actually followed. Similarly, we know that there are three active promoter binding sites in the extant *GAL1 *promoter, and three in the ancestral *GAL1/3 *promoter, so we assume that were no additional binding site gains and losses during the specialization of *GAL1/3 *to *GAL1*. Likewise, we know that the *GAL3 *promoter has a single binding site, so we assume that two sites in *GAL1/3 *were lost without any additional intervening binding site gains or losses. Finally, since there is little data from other species to estimate parameter changes in the GAL network, we have assumed that these either remained unchanged or have incurred small changes (see Methods for parameter perturbation experiments).

**Figure 3:**
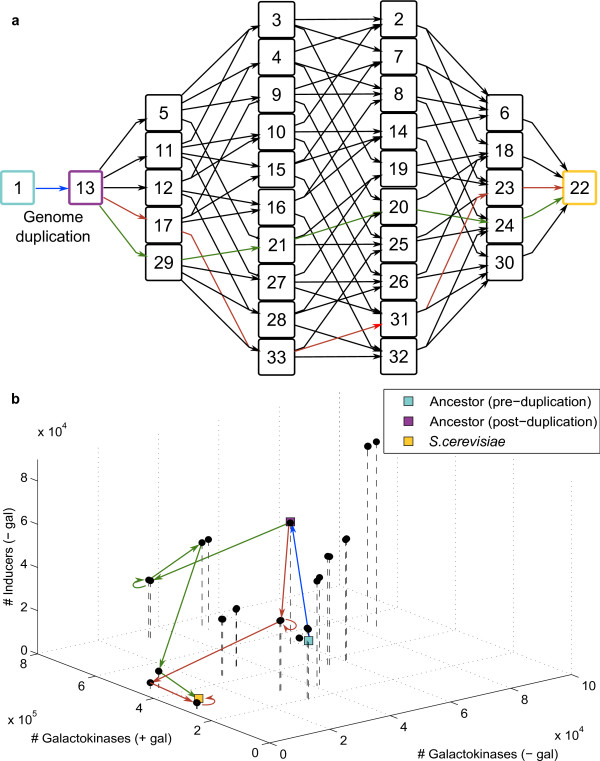
**Mapping of regulatory space to functional space**. **a**. GAL networks connecting the ancestor and intermediates to *S. cerevisiae*. Boxed numbers correspond to the network configurations in **Table 2**. Arrows indicate the connections between networks resulting from the possible evolutionary changes at each intermediate. Two example paths are shown. Red arrows indicate the sequence: specialization of *GAL3*, specialization of *GAL1*, loss of *GAL4 *duplicate, loss of *GAL2 *duplicate, loss of *GAL80 *duplicate; green arrows indicate the sequence: specialization of *GAL1*, loss of *GAL2 *duplicate, loss of *GAL80 *duplicate, specialization of *GAL3*, loss of *GAL4 *duplicate. The blue arrow indicates the WGD. **b**. Functional space of GAL network operation showing three network features. More galactokinase (Gal1p, Gal1/3p) molecules in high galactose (+ gal) indicate higher induction strength. Less galactokinase and co-inducer (Gal3p, Gal1/3p) molecules in very low galactose (- gal) indicate better repression strength and switch effectiveness respectively. Black spheres are intermediate GAL networks. *S. cerevisiae *and the pre- and post- WGD ancestors are indicated by colored squares. Red, green, and blue arrows indicate the evolutionary changes from **Figure 3a**.

We simulated all 33 of the possible network configurations that can arise as combinations of the regulatory changes required for the ancestral network configuration to evolve to the extant configuration in *S. cerevisiae *(1 pre-WGD ancestor + 1 post-WGD ancestor + 5 configurations with one event + 10 configurations with two events + 10 configurations with three events + 5 configurations with four events + 1 *S. cerevisiae*. See Table 2). These networks link *S*. *cerevisiae *and its ancestor via 120 unique evolutionary paths. Each path consists of 7 networks which are connected via the WGD event and a unique sequence of five evolutionary changes (Figure 3a).

**Table 2:** Configurations for the GAL network variants modeled in this study

	Gene copy-number					
**Network**	***GAL1***	***GAL2***	***GAL3***	***GAL4***	***GAL80***	***GAL1/3***

**1**	0	1	0	1	1	1

**2**	0	1	0	1	1	2

**3**	0	1	0	1	2	2

**4**	0	1	0	2	1	2

**5**	0	1	0	2	2	2

**6**	0	1	1	1	1	1

**7**	0	1	1	1	2	1

**8**	0	1	1	2	1	1

**9**	0	1	1	2	2	1

**10**	0	2	0	1	1	2

**11**	0	2	0	1	2	2

**12**	0	2	0	2	1	2

**13**	0	2	0	2	2	2

**14**	0	2	1	1	1	1

**15**	0	2	1	1	2	1

**16**	0	2	1	2	1	1

**17**	0	2	1	2	2	1

**18**	1	1	0	1	1	1

**19**	1	1	0	1	2	1

**20**	1	1	0	2	1	1

**21**	1	1	0	2	2	1

**22**	1	1	1	1	1	0

**23**	1	1	1	1	2	0

**24**	1	1	1	2	1	0

**25**	1	1	1	2	2	0

**26**	1	2	0	1	1	1

**27**	1	2	0	1	2	1

**28**	1	2	0	2	1	1

**29**	1	2	0	2	2	1

**30**	1	2	1	1	1	0

**31**	1	2	1	1	2	0

**32**	1	2	1	2	1	0

**33**	1	2	1	2	2	0

**Figure 4:**
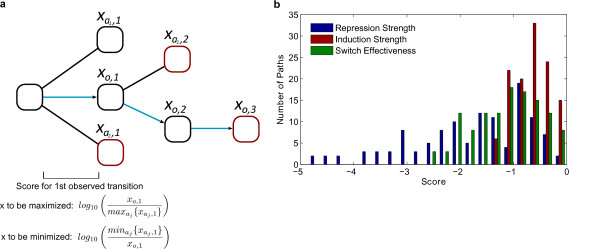
**Scoring the evolution of network features**. **a**. Schematic of the path scoring scheme. In this subset of networks (blocks) fold-changes in the feature to be scored are denoted by *x*. The observed networks in this path are connected by blue arrows and indexed with the *o *subscript. Networks not connected by these arrows are networks resulting from the available alternative evolutionary changes at each intermediate network, indexed with the *a *subscript. Red outlines indicate networks which optimize the feature at each stage. Each evolutionary change in a path is penalized by the logarithm of the ratio of the observed feature score to the best alternative feature score at each intermediate network. The total path score is the sum of the penalties for the five evolutionary changes (see Methods for a detailed explanation of the scoring scheme). **b**. Distribution of evolutionary path scores for the three scored features. Paths with higher scores perform better in optimizing that network feature.

Evolutionary changes, such as the WGD, gene duplicate loss, and specialization of *GAL1/3 *to *GAL1 *and *GAL3 *connect these networks along evolutionary trajectories to *S. cerevisiae *(see **Figure 4**).

To evaluate the quantitative function of a network we considered the number of protein molecules at equilibrium in very low galactose (10^-8^M, the "uninduced state") and conditions of very high galactose (0.1 M, the "induced state"). We defined three network features of interest for further examination:

Feature 1 "repression strength", is defined as the number of galactokinase molecules (Gal1p, Gal1/3p) in the uninduced state (10^-8 ^M galactose), such that a smaller number indicates better repression;

Feature 2 "induction strength", is defined as the number of galactokinase molecules in the induced state (0.1 M galactose), such that a larger number indicates stronger induction;

Feature 3 "switch effectiveness" is defined as the number of co-inducers (Gal3p, Gal1/3p) in the uninduced state (10^-8 ^M galactose), such that a smaller number indicates a more effective switch.

Changes in gene copy-number, protein function, and the number and arrangement of *cis-*regulatory sequences often resulted in significant changes in the network's quantitative function, as indicated by the differences in the steady-state galactokinase and co-inducer concentrations between the 33 network configurations (Figure 3b). The magnitude and direction of these changes, however, depended both on the particular evolutionary event being effected as well as on the configuration of the network being changed - that is, on the history of evolutionary changes that a network has accumulated. For example, we found that loss of the *GAL80 *repressor gene duplicate has a significantly smaller impact on repression strength if it occurs prior to the specialization of both copies of *GAL1/3 *(data not shown). This is because Gal80p-mediated repression of *GAL1 *is much stronger than that of *GAL1/3 *due to differences in the promoter regions of the two genes.

### Quantitative assessment of the order of evolutionary changes reveals that there are evolutionary paths that optimize specific network features

All of the evolutionary paths in the space of possible regulatory networks terminate at *S*. *cerevisiae *and, as a consequence, eventually accrue identical changes in function relative to the inferred ancestral network. Nevertheless, because the effect of an evolutionary change depends on the configuration of the network at that point in evolution, intermediate GAL networks leading to *S. cerevisiae *occupy markedly different regions in functional space depending on the sequence in which evolutionary changes accumulate.

For each of the 120 evolutionary paths we applied a scoring scheme (Figure 4a) which penalizes an evolutionary event at that intermediate if it does not result in the best possible change in the network feature being scored (see Methods for a detailed explanation of our scoring scheme). We find large variations in these scores (Figure 4b) confirming that different sequences of evolutionary changes lead to different quantitative network function in the evolutionary intermediates. Of the three features considered, induction strength shows the smallest variation, perhaps indicating that there is the least potential for evolution in this axis (see Discussion).

The path that optimizes repression strength (0 penalty) consists of the sequence: specialization of *GAL3*, specialization of *GAL1*, loss of *GAL4 *duplicate, loss of *GAL2 *duplicate, loss of *GAL80 *duplicate (red path in Figure 3a, b). The sequence: specialization of *GAL1*, specialization of *GAL3*, loss of *GAL4 *duplicate, loss of *GAL2 *duplicate, loss of *GAL80 *duplicate, optimizes switch effectiveness. The sequence: specialization of *GAL1*, loss of *GAL2*, loss of *GAL80*, specialization of *GAL3*, loss of *GAL4 *(green path in Figure 3a, b) optimizes induction strength.

**Figure 5:**
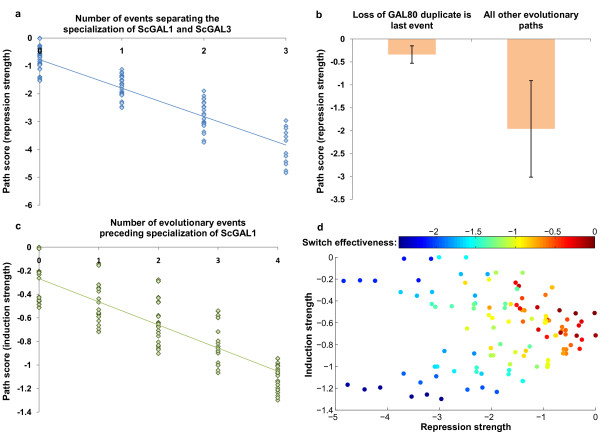
**Assessment of evolutionary paths**. **a**. Each data point represents an evolutionary path, plotted according to the number of evolutionary events separating the specialization of *GAL1 *and *GAL3 *and the switch effectiveness score. Scores improve as specialization events take place in closer succession (blue line indicates a linear regression, R^2 ^= 0.85). **b**. Bars are mean path scores (+/- standard deviation indicated by error bars) for switch effectiveness in paths where loss of the GAL80 duplicate is the last evolutionary change and paths where GAL80 duplicate loss is not the last change. **c**. Each data point represents an evolutionary path, plotted according to their induction strength scores and the number of evolutionary events preceding Gal1p specialization. Scores worsen as more evolutionary events precede the specialization of *GAL1 *(green line represents the linear regression, R^2 ^= 0.72). **d**. Data points are evolutionary paths, plotted according to the three scored network features. While there are paths that maximize both repression strength and switch effectiveness scores, it is not possible to simultaneously optimize either repression or effectiveness and induction strength in the same evolutionary path.

We found that evolutionary paths where the two specialization events of *GAL1/3 *to *GAL1 *and *GAL3 *occur in close sequence perform well in maximizing repression strength and switch effectiveness for intermediate GAL networks. Path scores in these two features are negatively correlated (R^2 ^= 0.85) with the number of evolutionary events separating the specialization of *GAL1 *and *GAL3 *(Figure 5a). In addition, paths where the second copy of *GAL80 *is lost after all other evolutionary changes show significantly higher repression strength compared to other paths (-0.34+/-0.19 vs. -1.96+/-1.05, *P *= 0.001, two-sample t-test, Figure 5b). It was also found that the group of paths where specialization of *GAL1/3 *takes place before any gene duplicates are lost has a higher average switch effectiveness score than paths where duplicates are lost prior to specialization (-0.16+/-0.12 vs. -1.24+/-0.57, *P *< 0.001, two-sample t-test, data not shown). Finally, we find a correlation (R^2 ^= 0.72) between how early *GAL1 *specializes and the strength of induction (Figure 5c), as well as a weaker correlation with how late *GAL3 *specializes and the induction strength scores (R^2 ^= 0.34, data not shown).

We next explored the relationship between the scores of the different features. Evolutionary paths tend to maximize switch effectiveness scores and repression strength scores together (R^2 ^= 0.72, data not shown). Interestingly, however, paths cannot optimize induction strength and switch effectiveness/repression strength simultaneously (Figure 5d). As a result, evolutionary paths may optimize switch effectiveness/repression or strength of induction, but not both features at the same time.

In the preceding sections, we have made the assumption that the parameters of the network remain the same as those observed or inferred in *S. cerevisiae*. However, in order to test whether the results presented above are sensitive to parameters, we have performed a series of perturbation experiments in which parameters were randomly modified during evolution and found that the observations presented were all recapitulated in the perturbed networks (see Methods).

## Discussion

Using a quantitative model of the regulatory network we were able integrate the wealth of experimental knowledge about the *S. cerevisiae *GAL network with promoter swap experiments and to infer and simulate the operation of an ancestral form of the GAL network. Two of our observations are consistent with the report of increased fitness after promoter specialization [21]. First, our observation of a higher expression of *GAL1 *relative to the ancestor could lead to an elevated metabolic capacity (galactose flux through the galactokinases) for *S. cerevisiae *in high galactose, and therefore perhaps to increased growth rate. Second, we observed a decrease in the number of protein molecules in the absence of galactose, which could lead to increased growth in the absence of galactose through a decrease in the energetic cost of protein synthesis (smaller number of protein molecules) (see [32] for an account of cost-benefit analysis of gene expression in a different system).

Despite the apparent consistency between our results and the report of adaptive specialization along the *S. cerevisiae *lineage, we found it difficult to establish a rigorous relationship between the quantitative function of the network and the network's fitness landscape. For example, in order to understand the potential benefit of increased levels of *GAL1 *expression, it is necessary to consider the impact of the galactokinase on the entire metabolic network [33]. In particular, consideration of the toxicity of the metabolic intermediates is important and greatly complicates this analysis [34]. Perhaps even more difficult is that we do not know the relative importance to the population of the induced or uninduced states of the GAL network. Clearly, the amount of time that *S. cerevisiae *may spend exposed to galactose depends on the environment and history of the population. It is unclear if this information will ever be available to us.

### Analysis of evolutionary paths

Our scoring of evolutionary paths in the functional space indicates that most potential phenotypic divergence takes place in the uninduced state (repression strength and switch effectiveness, Figure 5b). This is consistent with experimental findings that report a greater effect of *cis*-regulatory variation on gene repression than on induction [35]. We also found that no evolutionary path takes optimal steps (steps which incur no penalty) in every functional dimension at every network intermediate. In particular, we found that paths that maximized repression strength and switch effectiveness were sub-optimal with respect to induction strength.

Interestingly, we note that GAL networks in other extant species have specialized their *GAL1/3 *homologues, but have retained various duplicates. For example, *S. bayanus *and *S. castellii *have specialized forms of *GAL1 *and *GAL3*, but *S. bayanus *has kept two *GAL80 *copies and reacquired a tandem *GAL2 *duplicate [15], while *S. castellii *has kept two *GAL4 *and *GAL80 *copies [36]. According to our evolutionary path assessment, the specialization of *GAL1 *and *GAL3 *before the loss of regulatory gene duplicates, as well as the close proximity of these specialization events, improves switch effectiveness and repression strength. Retention of the *GAL80 *duplicate in both species is also consistent with better repression strength scores (Figure 4a, b).

We speculate a possible explanation for these observations. The GAL network allows for a larger phenotypic divergence in the uninduced state (consistent with our observations of larger variance in path scores pertaining to switch effectiveness and strength of repression). The network's capacity to improve and, therefore, the growth advantage, is greater if networks follow the path of uninduced state optimization.

### Assumptions of the approach

Our method of exploring the evolution of the GAL network is based on several assumptions. First, in constructing the evolutionary paths leading to *S*. *cerevisiae*, we assume that the evolutionary distance between two GAL network intermediates that are connected by a regulatory change is always constant - that is, we treat all evolutionary changes as equally likely to occur at each step of a path. Second, we make the assumption that regulatory elements not present in *S. cerevisiae *were also not present in the ancestor. While network features not present in *S. cerevisiae*, such as a unique UAS configuration on a gene promoter, could have existed in the ancestor or intermediate networks, we have constrained the network's regulatory space to include only those evolutionary events required under the assumption of maximum parsimony - which states that, when confronted with multiple evolutionary scenarios that explain some data, the scenario that requires the fewest evolutionary events is the most probable to have been followed [31]. Third, while we attempt to model parameter perturbations during network evolution, we cannot exclude the possibility that mutations can occur that have very large effects on parameter values. Potentially, experimental characterization of the GAL network in other species [37] could give an indication of such events, which could then be modeled as distinct evolutionary changes in the history of the GAL network.

## Conclusions

A hybrid stochastic-deterministic modeling framework has been used to explore the effect of regulatory divergence in the GAL network following the whole genome duplication. We have shown that some evolutionary paths optimize distinct features of network operation and that intermediate GAL networks in the lineage to *S. cerevisiae *could not have optimized all network features at the same time.

## Methods

### Hybrid stochastic-deterministic simulation algorithm

Our model of the GAL regulatory network contains 18 molecular species with extracellular galactose acting as the input to the system (Table 3). Simulations of the GAL network were performed using a hybrid stochastic-deterministic algorithm where each iteration of the algorithm consists of a stochastic part and a deterministic part (the latter of which may be skipped as an approximation method).

**Table 3:** Molecular species used in the GAL network models

Name	Description
*Gal*1*p*	Galactokinase

*Gal*2*p*	Galactose permease

*Gal*3*p_i_*	Co-inducer (inactive)

*Gal*3*p_a_*	Co-inducer (active)

*Gal*4*p*	Transcriptional activator

*Gal*80*p*	Transcriptional repressor

*Gal*1/3*p_i_*	Bi-functional galactokinase/inducer (inactive)

*Gal*1/3*p_a_*	Bi-functional galactokinase/inducer (active)

*Gal*3-80*p*	Inducer-repressor complex

*Gal*1/3-80*p*	Galactokinase/inducer-inhibitor complex

*Gal*4-80*p*	Activator-repressor complex

*GAL*1	Encodes for Gal1p

*GAL*2	Encodes for Gal2p

*GAL*3	Encodes for Gal3p

*GAL*4	Encodes for Gal4p

*GAL*80	Encodes for Gal80p

*GAL*1/3	Ancestral gene. Encodes for Gal1/3p

*Gal_in_*	Intracellular galactose

#### 1. Deterministic part

The first part of the algorithm begins with an algebraic step which models galactose transport to the cell interior:

$$\left[gal_{in}\right]=\left[gal_{out}\right]\left(a+b\cdot gal2p\right),$$

where we used *a *= 0.001 and *b *= 0.01 as transport coefficients for the non-specific hexose transporters and galactose permeases (Gal2p) respectively. In the above equation, and in equations that follow, molecular names in square brackets refer to the concentration of that species, whereas names without brackets refer to that species' number of molecules.

Protein-galactose and protein-protein interactions involved in signal transduction are modeled as a system of ODEs:

$$\frac{\partial }{\partial t}\left[Gal\right]=M\left[Gal\right],$$

***M ***is the mass matrix specified by the following equations:

$$\left[gal_{in}\right]+\left[Gal3p_{i}\right]\overset{k_{3}^{+}}{\underset{k_{3}^{-}}{⇌}}\left[Gal3p_{a}\right],$$

$$k_{3}^{+}=10,k_{3}^{-}=1$$

$$\begin{array}{c} \left[gal_{in}\right]+\left[Gal13p_{i}\right]\overset{k_{13}^{+}}{\underset{k_{13}^{-}}{⇌}}\left[Gal13p_{a}\right],\\ k_{13}^{+}=10,k_{13}^{-}=1 \end{array}$$

$$\begin{array}{c} \left[Gal3p_{a}\right]+\left[Gal80p\right]\overset{k_{380}^{+}}{\underset{k_{380}^{-}}{⇌}}\left[Gal380p_{a}\right],\\ k_{380}^{+}=10,k_{380}^{-}=1 \end{array}$$

$$\begin{array}{c} \left[Gal13p_{a}\right]+\left[Gal80p\right]\overset{k_{1380}^{+}}{\underset{k_{1380}^{-}}{⇌}}\left[Gal1380p_{a}\right],\\ k_{1380}^{+}=10,k_{1380}^{-}=1 \end{array}$$

$$\begin{array}{c} \left[Gal80p\right]+\left[Gal4p\right]\overset{k_{480}^{+}}{\underset{k_{480}^{-}}{⇌}}\left[Gal480p\right],\\ k_{480}^{+}=1,k_{480}^{-}=1 \end{array}$$

Where the *k^+ ^*and *k^- ^*are the forward and reverse kinetic rates respectively for each reaction. Steady-state concentrations are used to update the components of the system, but simulation time is not advanced. This rule permits us to treat protein-protein/galactose interactions as much faster reactions than protein synthesis/degradation (approximately instantaneous), while still allowing stochastic fluctuations to influence the deterministic solution trajectory in this multi-stable system (data not shown).

#### 2. Stochastic part

In the second part of the algorithm, the processes of protein synthesis and degradation are modeled stochastically. The stoichiometric matrix was constructed from the following reactions:

$$\begin{array}{c} GAL_{i}\overset{s_{i}}{\to }GAL_{i}+b\;\cdot \;Gal_{i'}\\ i\in \left\{1,2,3,4,80,13\right\}\\ (\text{protein synthesis, burst factor, }b=\text{2}\text{.5),} \end{array}$$

$$\begin{array}{c} Gal_{i}\overset{d_{i}}{\to }Gal_{i}-1,\\ i\in \left\{1,2,3_{i},3_{a},380,4,480,80,13_{i},13_{a},1380\right\}\\ (\text{protein degradation),} \end{array}$$

for a total of 17 reaction channels involving 18 molecular species. Stochastic simulations were performed using the Gillespie algorithm [38]. Transcription and translation were modeled as a single step, where each mRNA molecule generates 2.5 protein molecules. The propensity function, *s*, for transcribing an mRNA molecule is

$$\begin{array}{c} s_{i}=GAL_{i}\cdot f_{p_{i}}(\left[Gal4,Gal480\right]),\\ i\in \left\{1,2,3,4,80,13\right\}, \end{array}$$

where $f_{P_{i}}$ is the promoter model assigned to gene *GAL_i _*(see Promoter models section). Our model accounts for changes in gene copy-number because the probability of transcript production is proportional to the number of gene copies for that molecular species in the network. This is a result of the stochastic simulation algorithm, where multiplying the promoter function by gene number is equivalent to introducing additional identical reaction channels. The propensity function for protein degradation, *d*, is

$$\begin{array}{c} d_{i}=Gal_{i}\cdot \gamma ,\\ i\in \left\{1,2,3,3_{i},3_{a},380,4,480,13_{i},13_{a},1380\right\},\\ \gamma =0.002, \end{array}$$

where *γ *is the degradation rate.

The Gillespie algorithm samples the joint probability distribution of reaction events and times at each iteration of the stochastic part, selects a reaction, and generates a time interval containing no reactions, *τ*, which is used to advance the simulation time.

### Promoter models

The propensity function of protein synthesis in the stochastic part of the simulation algorithm is driven by a physicochemical promoter model. An upstream activation sequence (UAS) on a promoter may be empty, occupied by a Gal4p molecule, or occupied by a Gal4-80p molecule. Sites occupied by Gal4p interact with and recruit RNA polymerase and the transcriptional machinery with an affinity that is intrinsic to the particular promoter (Table 4). Empty sites and sites occupied by Gal4-80p may also allow some basal transcription to occur. The promoter function, *f*_PI_, for transcription is the sum of the contribution of all sites to transcription initiation in each occupancy configuration, weighted by the configuration's probability:

**Table 4:** GAL promoter models

Promoter	# UAS	Cooperative UAS pairs	Contribution to transcript production per site
*GAL*1	3	2	[-0.05,21.00, -0.05]

*GAL*2	2	1	[-0.01,16.00, -0.01]

*GAL*3	1	0	[0.14,9.00,0.14]

*GAL*4	0	0	Constitutive expression: 0.06

*GAL*80	1	0	[0.01,2.00,0.01]

*GAL*1/3	3	0	[0.40,17.70,0.40]

# UAS indicates the number of binding sites that may be empty, bound by Gal4p, or bound by Gal4-80p. UAS pairs that are appropriately spaced to permit cooperative interactions between adjacently bound proteins are listed for each promoter. For each promoter, the strength of transcription initiation for each site that is either: [empty, bound by Gal4p, bound by Gal4-80p] is given in square brackets. A negative number indicates that transcript initiation is repressed by the occupancy state of the UAS. Note that in spite of the approximately 50 base pair separation between the two UAS in *GAL2 *we have allowed cooperative protein binding (see [5]). We also do not consider the 4^th ^UAS on *GAL1 *(and *GAL1/3*) after indications that it may not be as important for gene expression as the other UAS [42].

$$f_{P_{i}}(\left[Gal4\right],\left[Gal480\right])=\frac{{\sum_{c}K_{c}\left[Gal4\right]}^{n4_{c}}{\left[Gal480\right]}^{n480_{c}}e^{\frac{1}{\beta }\Delta G_{c}}}{\sum {\left[Gal4\right]}^{n4_{c}}{\left[Gal480\right]}^{n480_{c}}e^{\frac{1}{\beta }\Delta G_{c}}}$$

Where *c *index the occupancy configurations for the promoter driving gene *GAL_i_*. *K_c _*is the sum of contributions to transcription initiation by all sites in configuration *c*. *n*4*_c _*and *n*480_c _are the numbers of Gal4p- and Gal480p- bound sites in configuration *c *respectively. *β *(1.68 kcal/mol) is the product of the gas constant and the system's temperature (300 K). Δ*G_c _*is the sum of the Gibbs' free energy changes from all DNA-protein binding events in configuration *c*, as well as any cooperative interactions between adjacently bound activator and/or repressor proteins which stabilize the DNA-protein complex. Taking the configuration where all sites are empty as the reference state of each promoter, we used -13.86 kcal/mol as the energy change for the binding of either a Gal4p or Gal4-80p to a UAS [39]. We chose -2.00 kcal/mol and -3.00 kcal/mol for energy changes due to a single cooperative interaction between appropriately positioned Gal4p and Gal480p respectively because they led to proper quantitative promoter function in *S*. *cerevisiae*, and we could not identify these parameters in the literature. In the absence of any UAS on a promoter, as in the case for the GAL4 promoter, the propensity function evaluates to a constant, *Kc*, giving constitutive expression.

### Algorithm execution

The model was implemented and simulated in MATLAB^® ^(The MathWorks). Simulations were run for 8,000 seconds, which was found to be much longer than the typical time for equilibrium (data not shown). To decrease computation time - particularly in systems with high protein concentrations - the deterministic step was executed only every *n *iterations, where *n *scaled linearly with the total number of molecules participating in the deterministic step. The rationale behind this approximation is that stochastic synthesis or degradation of a few molecules will not significantly change the equilibrium of interacting proteins when large concentrations of proteins are already reacting. Trial simulations run with and without this approximation procedure followed similar induction dynamics, attained equilibrium at the same times, and had similar equilibrium behavior (data not shown).

Conversions between number of molecules and concentrations are performed at each step as required using 1.66 × 10^-15 ^and 23 × 10^-15 ^*L *as nuclear and cellular volumes respectively [40,41].

The mean and variance for the number of molecules for each species was obtained from the equilibrium distributions of 100 independent simulations at 8 extracellular galactose concentrations (10^-8^, 10^-7^, 10^-6^, 10^-5^, 10^-4^, 10^-3^, 10^-2^, and 10^-1 ^M). This was done for each of the 33 GAL networks to construct their response curves to increasing galactose concentrations.

### Scoring scheme for evolutionary paths

Each evolutionary event is scored according to its effect on some feature - for example, the number of molecules with galactokinase activity at a certain extracellular galactose concentration. We begin scoring evolutionary events after the genome duplication and consider the 120 unique combinations of the five events required for the post-genome duplication ancestor to evolve to *S. cerevisiae*. For each feature to be scored, we defined either the largest fold-increase or fold-decrease to be the optimal change (see Results section). Events are scored relative to the possible alternative events at each intermediate network along the path. An observed event is penalized if it does not result in the best possible change in the feature from amongst the possible events. To keep the scoring scheme consistent, if the feature is to be maximized, we used:

$$s=\sum_{i=1}^{5}\log_{10}\left(\frac{x_{a=o,i}}{\max_{\text{a}}\left\{x_{{\text{a}}_{\text{j}}\text{,i}}\right\}}\right)$$

If the feature is to be minimized, the total path score is:

$$s=\sum_{i=1}^{5}\log_{10}\left(\frac{\min_{\text{a}}\left\{x_{a_{j}}\right\}}{x_{a=o,i}}\right)$$

where *x*_*a,i*_, is the fold-change in the network feature resulting from the *i*-th evolutionary step after the genome duplication, for the *a*-th alternative network. We indicate the observed evolutionary choice in the path at step *i*, as *a *= *o*. See Figure 4a for an illustration of the scoring scheme.

The scoring scheme provides an objective means of ranking evolutionary paths in regulatory space that optimize some network feature. The path with the highest possible score (which is 0) consists of the order of events which results in the best possible sequence of changes in the scored feature. Conversely, the worst-scoring path follows none of the best alternatives (except for the last evolutionary event).

### Parameter selection

The number of protein molecules for each molecular species were inferred or taken directly from various sources as indicated in Table 1. In addition to fold-induction data, the following gene expression relationships from promoter replacement studies in *S. cerevisiae *[21] were used:

$$\begin{array}{c} PScGAL3\to GAL1=\\ 2.12\times PScGAL1\to GAL1,\\ \text{uninduced conditions} \end{array}$$

$$\begin{array}{c} PScGAL3\to GAL1=\\ 0.13\times PScGAL1\to GAL1,\\ \text{induced conditions} \end{array}$$

$$\begin{array}{c} PKlacGAL1/3\to GAL1=\\ 9.95\times PScGAL1\to GAL1,\\ \text{uninduced conditions} \end{array}$$

$$\begin{array}{c} PKlacGAL1/3\to GAL1=\\ 0.54\times PScGAL1\to GAL1,\\ \text{induced conditions} \end{array}$$

*PGALi→GAL*1 indicates a network where *GAL*1 is driven by *GALi*'s wild-type promoter, in a *gal*1 - Δ background. *PScGALi *is the *S. cerevisiae *wild type promoter for *GALi *and *PKlacGAL1/3 *is the *K. lactis *wild type promoter for *GAL*1/3, which is thought to be similar to the ancestral bifunctional gene promoter.

The relationship [*Gal3p*] ≈ 5[*Gal80p*] in both uninduced and induced conditions was also implemented [6]. These parameters were used to simulate all networks in the regulatory space that we have investigated - under the assumption that, while regulatory features might evolve, their mode of operation remains unchanged (see Discussion).

### Parameter set perturbations

To investigate the robustness of our results to perturbations in the parameter set we executed 50 further simulations of each of the 120 possible evolutionary paths while enforcing a random, non-persistent, parameter change at each network intermediate, not including *S. cerevisiae*. Specifically, we allowed random perturbations in the following parameters and ranges:

1. Kinetic parameters: Multiplicative factor in [0.1, 10] from 10*^U(-1,1)^*.

2. Degradation rates, burst factor: Multiplicative factor in [0.5, 2] from 2*^U(-1,1)^*.

3. Contributions to transcript production (promoter models): Multiplicative factor in [0.5, 2] from 2*^U(-1,1)^_._*

4. Binding energies (promoter models): Additive factor in [-1, 1] from *U(-1,1)*.

where *U *is the uniform distribution.

These random parameter perturbations can be thought of as minor mutations in the network components which do not form part of the parsimonious set of evolutionary changes along the lineage to *S. cerevisiae*, but which may nevertheless have occurred and subsequently been lost.

Under parameter perturbations, evolutionary path score distributions had larger variance, often skewed toward worse scores, with occasional perturbations completely abolishing the switch- like behavior of the system (Additional File 1, **Figures S1-S3**). Most perturbations, however, did not have a large impact on perturbed path scores.

Specifically, we find that the number of evolutionary events separating the specialization of ScGAL1 and ScGAL3 again correlates negatively with repression strength scores (R^2 ^= 0.85, Additional File 1, **Figure S4a**). The number of events preceding the specialization of ScGAL1 correlates negatively with induction strength scores (R^2 ^= 0.64, Additional File 1, **Figure S4b**). A significant difference in repression strength scores was again found between evolutionary paths were GAL80 duplicate loss is the last event and all other paths (-0.40 +/- 0.23 versus -2.08 +/- 1.03, P < 0.01, Additional File 1, **Figure S4c**). Perturbed evolutionary paths tend to maximize switch effectiveness scores and repression strength scores together (R^2 ^= 0.73, data not shown). Finally, we report that, even with a perturbed parameter set, there exists no single path which optimizes all three aspects of quantitative network behavior (Additional File 1, **Figure S4d**).

## Authors' contributions

AMM conceived of the project. AMM and CJ designed research and wrote the manuscript. CJ performed all research. Both authors have read and approved the final manuscript.

## Supplementary Material

Additional file 1 **Path score distribution comparisons and assessment of evolutionary paths under parameter perturbations**. Box plot comparisons of the three evolutionary path score distributions between unperturbed and perturbed paths (Figures S1-3). Correlations between evolutionary path scores and classes of paths under parameter perturbations (Figure S4). Click here for file

## References

[B1] Wyrick JJ Young RA Deciphering gene expression regulatory networks Curr Opin Genet Dev 2002 12 130 136 10.1016/S0959-437X(02)00277-0 11893484

[B2] Chua G Robinson MD Morris Q Hughes TR Transcriptional networks: reverse-engineering gene regulation on a global scale Curr Opin Microbiol 2004 7 638 646 10.1016/j.mib.2004.10.009 15556037

[B3] Lee TI Rinaldi NJ Robert F Odom DT Bar-Joseph Z Gerber GK Hannett NM Harbison CT Thompson CM Simon I Zeitlinger J Jennings EG Murray HL Gordon DB Ren B Wyrick JJ Tagne JB Volkert TL Fraenkel E Gifford DK Young RA Transcriptional regulatory networks in *Saccharomyces cerevisiae* Science 2002 298 799 804 10.1126/science.1075090 12399584

[B4] Teichmann SA Babu MM Gene regulatory network growth by duplication Nat Genet 2004 36 492 496 10.1038/ng1340 15107850

[B5] Lohr D Venkov P Zlatanova J Transcriptional regulation in the yeast GAL gene family: a complex genetic network FASEB J 1995 9 777 787 7601342 10.1096/fasebj.9.9.7601342

[B6] Rubio-Texeira M A comparative analysis of the GAL genetic switch between not-so distant cousins: *Saccharomyces cerevisiae *versus *Kluyveromyces lactis* FEMS Yeast Res 2005 5 1115 1128 10.1016/j.femsyr.2005.05.003 16014343

[B7] Traven A Jelicic B Sopta M Yeast Gal4: a transcriptional paradigm revisited EMBO Rep 2006 7 496 499 10.1038/sj.embor.7400679 16670683 PMC1479557

[B8] Ideker T Thorsson V Ranish JA Christmas R Buhler J Eng JK Bumgarner R Goodlett DR Aebersold R Hood L Integrated genomic and proteomic analyses of a systematically perturbed metabolic network Science 2001 292 929 934 10.1126/science.292.5518.929 11340206

[B9] Acar M Becskei A van Oudenaarden A Enhancement of cellular memory by reducing stochastic transitions Nature 2005 435 228 232 10.1038/nature03524 15889097

[B10] Ruhela A Verma M Edwards JS Bhat PJ Bhartiya S Venkatesh KV Autoregulation of regulatory proteins is key for dynamic operation of GAL switch in *Saccharomyces cerevisiae* FEBS Lett 2004 576 119 126 10.1016/j.febslet.2004.09.001 15474022

[B11] Ramsey SA Smith JJ Orrell D Marelli M Petersen TW deAtauri P Bolouri H Aitchison JD Dual feedback loops in the GAL regulon suppress cellular heterogeneity in yeast Nat Genet 2006 38 1082 1087 10.1038/ng1869 16936734

[B12] Hittinger CT Goncalves P Sampaio JP Dover J Johnston M Rokas A Remarkably ancient balanced polymorphisms in a multi-locus gene network Nature 2010 464 54 58 10.1038/nature08791 20164837 PMC2834422

[B13] Martchenko M Levitin A Hogues H Nantel A Whiteway M Transcriptional rewiring of fungal galactose-metabolism circuitry Curr Biol 2007 17 1007 1013 10.1016/j.cub.2007.05.017 17540568 PMC3842258

[B14] Slot JC Rokas A Multiple GAL pathway gene clusters evolved independently and by different mechanisms in fungi Proc Natl Acad Sci USA 2010 107 10136 10141 10.1073/pnas.0914418107 20479238 PMC2890473

[B15] Hittinger CT Rokas A Carroll SB Parallel inactivation of multiple GAL pathway genes and ecological diversification in yeasts Proc Natl Acad Sci USA 2004 101 14144 14149 10.1073/pnas.0404319101 15381776 PMC521130

[B16] Wolfe KH Shields DC Molecular evidence for an ancient duplication of the entire yeast genome Nature 1997 387 708 713 10.1038/42711 9192896

[B17] Kellis M Birren BW Lander ES Proof and evolutionary analysis of ancient genome duplication in the yeast *Saccharomyces cerevisiae* Nature 2004 428 617 624 10.1038/nature02424 15004568

[B18] Meyer J Walker-Jonah A Hollenberg CP Galactokinase encoded by GAL1 is a bifunctional protein required for induction of the GAL genes in *Kluyveromyces *lactis and is able to suppress the gal3 phenotype in *Saccharomyces cerevisiae* Mol Cell Biol 1991 11 5454 5461 1922058 10.1128/mcb.11.11.5454-5461.1991 PMC361914

[B19] Johnston M Davis RW Sequences that regulate the divergent GAL1-GAL10 promoter in *Saccharomyces cerevisiae* Mol Cell Biol 1984 4 1440 1448 6092912 10.1128/mcb.4.8.1440-1448.1984 PMC368932

[B20] Bajwa W Torchia TE Hopper JE Yeast regulatory gene GAL3: carbon regulation; UASGal elements in common with GAL1, GAL2, GAL7, GAL10, GAL80, and MEL1; encoded protein strikingly similar to yeast and *Escherichia coli *galactokinases Mol Cell Biol 1988 8 3439 3447 3062381 10.1128/mcb.8.8.3439-3447.1988 PMC363581

[B21] Hittinger CT Carroll SB Gene duplication and the adaptive evolution of a classic genetic switch Nature 2007 449 677 681 10.1038/nature06151 17928853

[B22] Shea MA Ackers GK The OR control system of bacteriophage lambda. A physical chemical model for gene regulation J Mol Biol 1985 181 211 230 10.1016/0022-2836(85)90086-5 3157005

[B23] Buchler NE Gerland U Hwa T On schemes of combinatorial transcription logic Proc Natl Acad Sci USA 2003 100 5136 5141 10.1073/pnas.0930314100 12702751 PMC404558

[B24] Bolouri H Computational Modelling of Gene Regulatory Networks - A Primer 2008 New Haven: Imperial College Press

[B25] Alfonsi A Cances E Turinici G Ventura BD Huisinga W Exact simulation of hybrid stochastic and deterministic models for biochemical systems ESAIM Proc 2005 14 113

[B26] Kiehl TR Mattheyses RM Simmons MK Hybrid simulation of cellular behavior Bioinformatics 2004 20 316 322 10.1093/bioinformatics/btg409 14960457

[B27] Rudiger S Shuai JW Huisinga W Nagaiah C Warnecke G Parker I Falcke M Hybrid stochastic and deterministic simulations of calcium blips Biophys J 2007 93 1847 1857 10.1529/biophysj.106.099879 17496042 PMC1959544

[B28] Salis H Kaznessis Y Accurate hybrid stochastic simulation of a system of coupled chemical or biochemical reactions J Chem Phys 2005 122 54103 10.1063/1.1835951 15740306

[B29] McAdams HH Arkin A Stochastic mechanisms in gene expression Proc Natl Acad Sci USA 1997 94 814 819 10.1073/pnas.94.3.814 9023339 PMC19596

[B30] Arkin A Ross J McAdams HH Stochastic kinetic analysis of developmental pathway bifurcation in phage lambda-infected *Escherichia coli *cells Genetics 1998 149 1633 1648 9691025 10.1093/genetics/149.4.1633 PMC1460268

[B31] Fitch WM Toward defining the course of evolution: minimum change for a specific tree topology Systematic Zoology 1971 20 4 406 416 10.2307/2412116

[B32] Dekel E Alon U Optimality and evolutionary tuning of the expression level of a protein Nature 2005 436 588 592 10.1038/nature03842 16049495

[B33] Ostergaard S Olsson L Nielsen J In vivo dynamics of galactose metabolism in *Saccharomyces cerevisiae*: metabolic fluxes and metabolite levels Biotechnol Bioeng 2001 73 412 425 10.1002/bit.1075 11320512

[B34] de Jongh WA Bro C Ostergaard S Regenberg B Olsson L Nielsen J The roles of galactitol, galactose-1-phosphate, and phosphoglucomutase in galactose-induced toxicity in Saccharomyces cerevisiae Biotechnol Bioeng 2008 101 317 326 10.1002/bit.21890 18421797

[B35] Bram RJ Lue NF Kornberg RD A GAL family of upstream activating sequences in yeast: roles in both induction and repression of transcription EMBO J 1986 5 603 608 3011415 10.1002/j.1460-2075.1986.tb04253.x PMC1166805

[B36] Rokas A Hittinger CT Transcriptional rewiring: the proof is in the eating Curr Biol 2007 17 R626 628 10.1016/j.cub.2007.06.025 17714646

[B37] Pannala VR Bhartiya S Venkatesh KV Experimental and steady-state analysis of the GAL regulatory system in *Kluyveromyces lactis* FEBS J 2010 277 2987 3002 10.1111/j.1742-4658.2010.07708.x 20528923

[B38] Gillespie DT Exact stochastic simulation of coupled chemical reactions J Phys Chem 1977 81 2340 2361 10.1021/j100540a008

[B39] Melcher K Xu HE Gal80-Gal80 interaction on adjacent Gal4p binding sites is required for complete GAL gene repression EMBO J 2001 20 841 851 10.1093/emboj/20.4.841 11179228 PMC145427

[B40] Anders A Lilie H Franke K Kapp L Stelling J Gilles ED Breunig KD The galactose switch in *Kluyveromyces lactis *depends on nuclear competition between Gal4 and Gal1 for Gal80 binding J Biol Chem 2006 281 29337 29348 10.1074/jbc.M604271200 16867978

[B41] Winey M Yarar D Giddings TH Mastronarde DN Nuclear pore complex number and distribution throughout the *Saccharomyces cerevisiae *cell cycle by three-dimensional reconstruction from electron micrographs of nuclear envelopes Mol Biol Cell 1997 8 2119 2132 9362057 10.1091/mbc.8.11.2119 PMC25696

[B42] Giniger E Ptashne M Cooperative DNA binding of the yeast transcriptional activator GAL4 Proc Natl Acad Sci USA 1988 85 382 386 10.1073/pnas.85.2.382 3124106 PMC279552

