# Supplementary material for: Modeling the evolution of a classic genetic switch
Source: BMC Syst Biol. 2011 Feb 5;5:24. doi: 10.1186/1752-0509-5-24 (PMC3048525; doi:10.1186/1752-0509-5-24)
Supplement: Additional file 1 — Path score distribution comparisons and assessment of evolutionary paths under parameter perturbations. Box plot comparisons of the three evolutionary path score distributions between unperturbed and perturbed paths (Figures S1-3). Correlations between evolutionary path scores and classes of paths under parameter perturbations (Figure S4). [file 1752-0509-5-24-S1.PDF]

Additional File 1

Supplementary Figures 1-3

Box plot comparisons of the three evolutionary path score distributions between unperturbed (red) and perturbed (blue) paths. Each of the 120 pairs of distributions has been obtained from 50 simulations allowed to reach steady-state for each of the GAL networks in that path, with or without random parameter perturbations. The paths were scored in a similar manner to the unperturbed path simulations (see Methods). Extreme values from parameter perturbations that abolish the network's switch-like behavior are clipped to the dotted lines in each graph, to allow better visual comparison of the distributions.

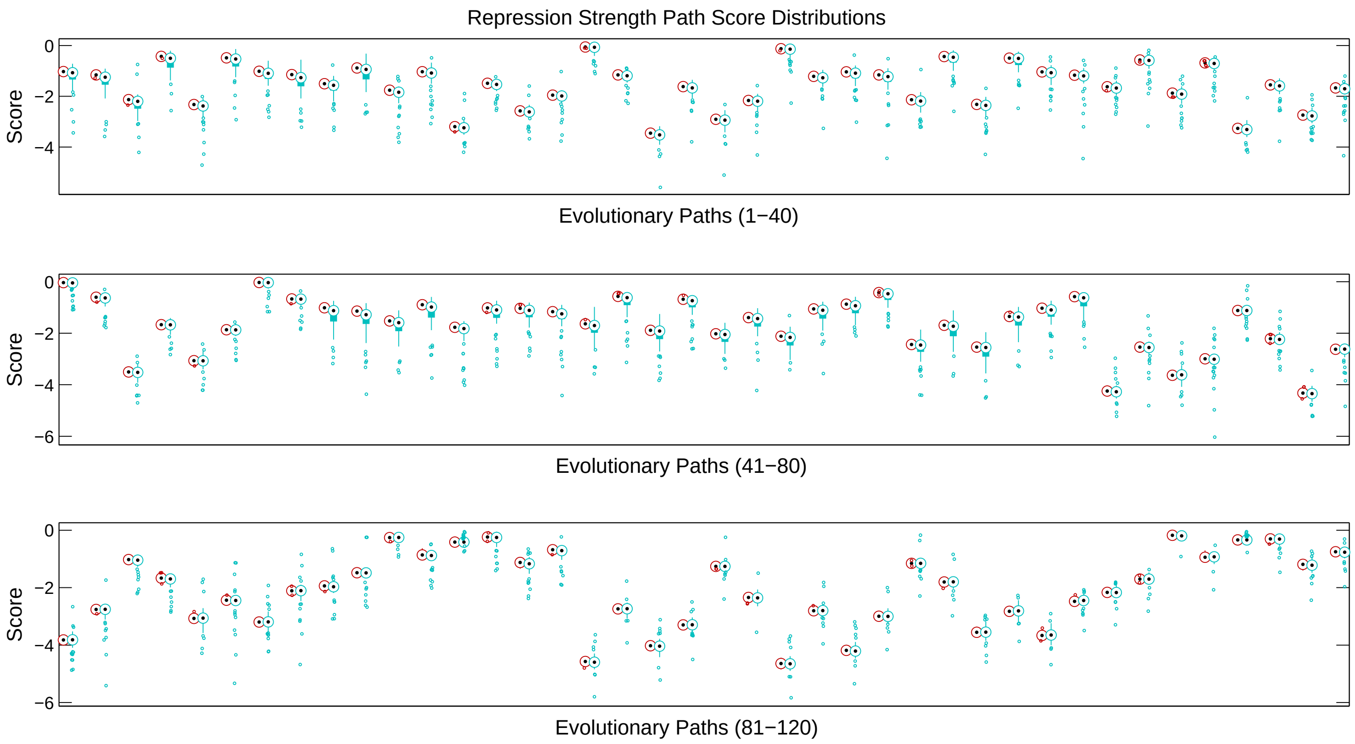

Figure S1: Repression strength score comparisons.

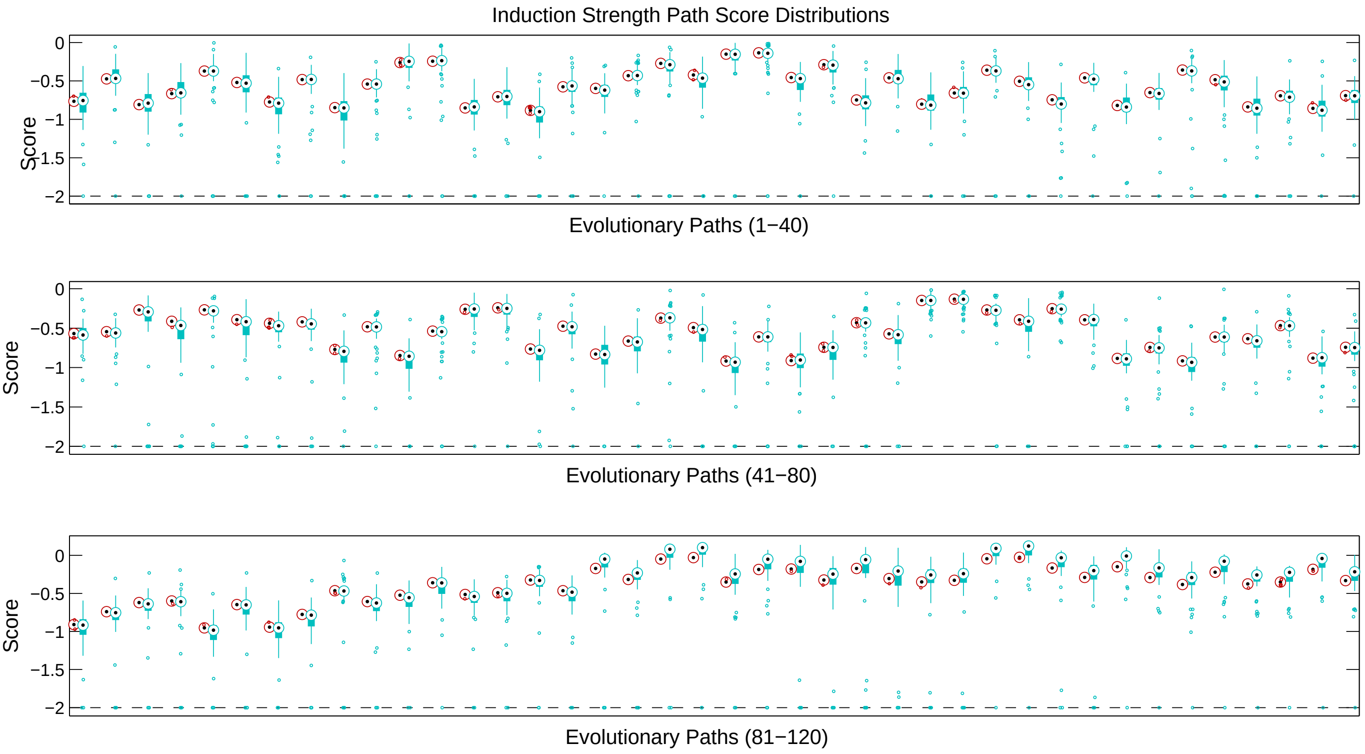

Figure S2: Induction strength score comparisons.

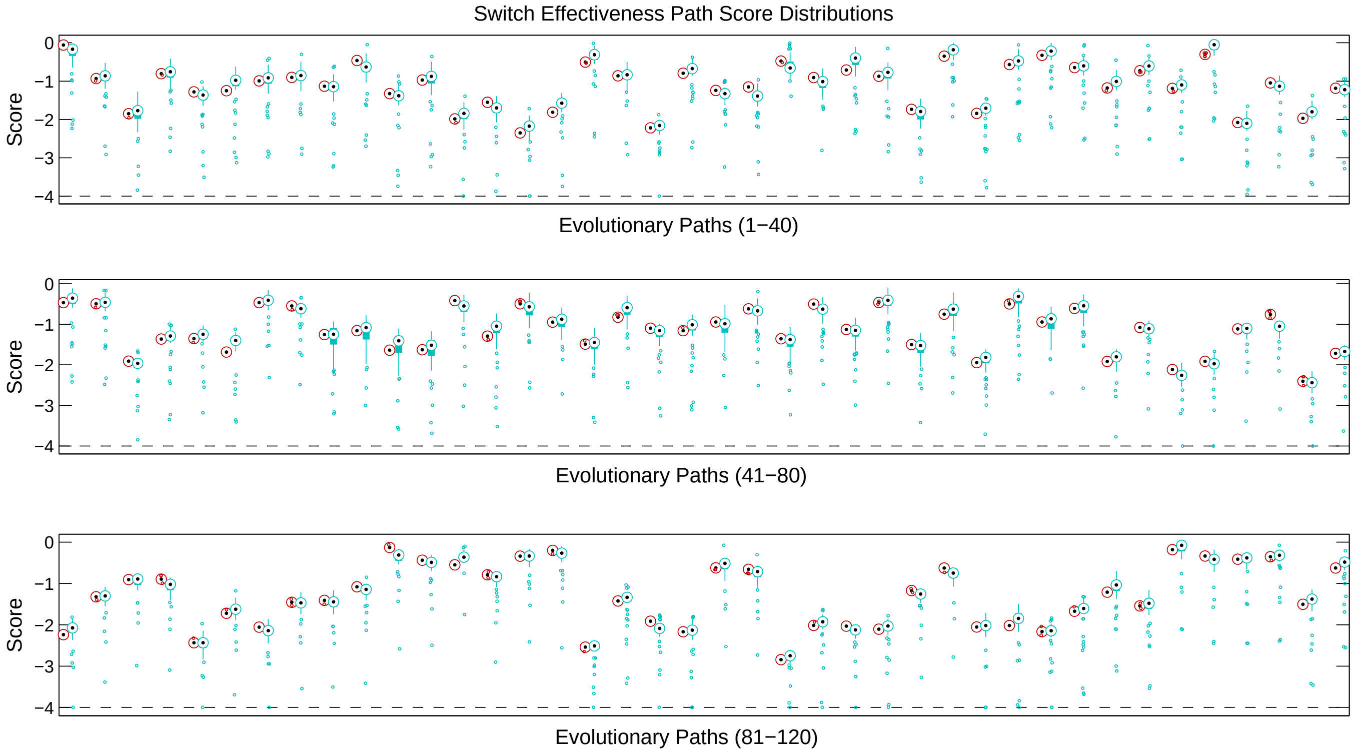

Figure S3: Switch effectiveness score comparisons.

Supplementary Figure 4

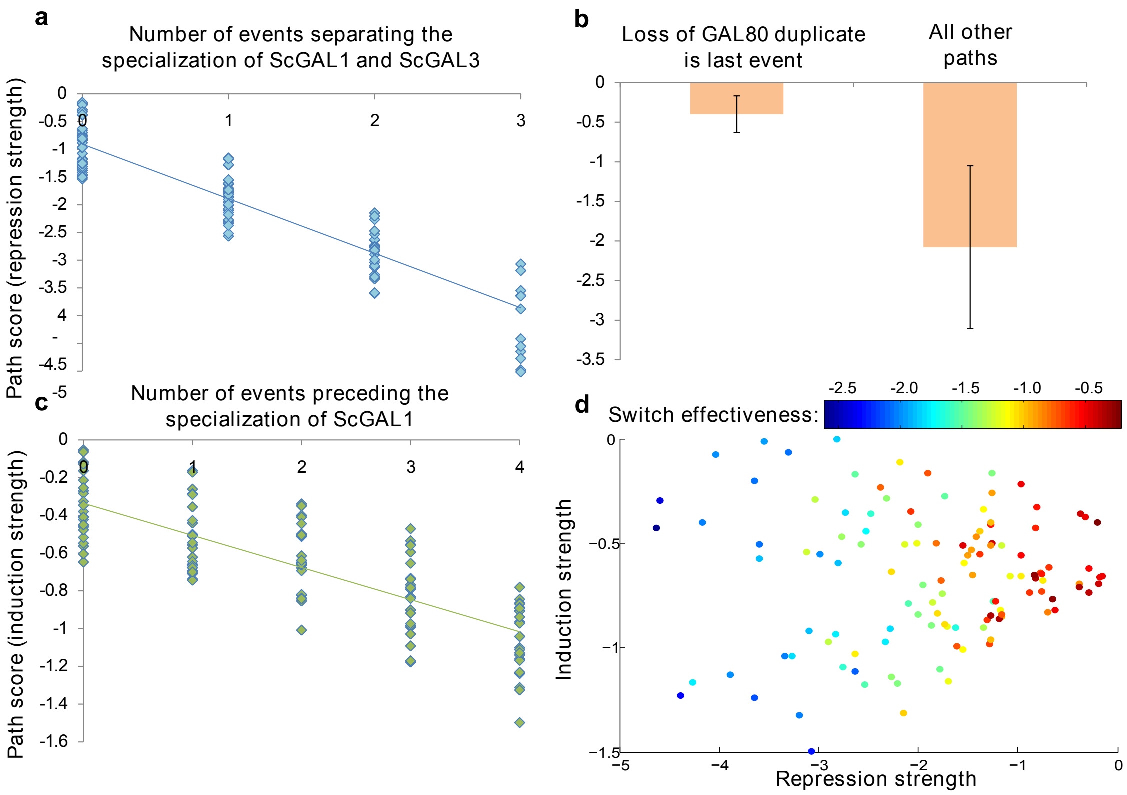

Figure S4: Assessment of evolutionary paths under parameter perturbations **a**. Each data point represents a perturbed evolutionary path, plotted according to the number of evolutionary events separating the specialization of GAL1 and GAL3 and the switch effectiveness score. Scores improve as specialization events take place in closer succession (blue line indicates a linear regression, R^2 = 0.85). **b**. Bars are mean perturbed path scores (+/- standard deviation indicated by error bars) for switch effectiveness in paths where loss of the GAL80 duplicate is the last evolutionary change and paths where GAL80 duplicate loss is not the last change. **c**. Each data point represents a perturbed evolutionary path, plotted according to their induction strength scores and the number of evolutionary events preceding Gal1p specialization. Scores worsen as more evolutionary events precede the specialization of GAL1 (green line represents the linear regression, R^2 = 0.64). **d**. Data points are perturbed evolutionary paths, plotted according to the three scored network features. While there are paths that maximize both repression strength and switch effectiveness scores, it is not possible to simultaneously optimize either repression or effectiveness and induction strength in the same evolutionary path.
